# Designed Synthesis of Nanostructured Materials as the Heterogeneous Catalysts

**DOI:** 10.3390/nano12244374

**Published:** 2022-12-08

**Authors:** Kyungsu Na

**Affiliations:** Department of Chemistry, Chonnam National University, Gwangju 61186, Republic of Korea; kyungsu_na@chonnam.ac.kr

The development of nanoscale syntheses and innovative characterization tools resulted in the tailored design of nanostructured materials with versatile abilities in many applications. Various types of engineered nanostructures, usually in the form of metal nanoparticles or nanoporous metal oxides, have been synthesized for various catalytic applications. The resultant solid-state-nanostructured materials can be used as heterogeneous catalysts that exhibit good stability and recyclability with repeated catalytic performances without significant changes in the intrinsic performances. In addition, the nanoscale surface control can control the surface property, affect the reaction process, and, finally, can change the product selectivity.

In this respect, the present Special Issue aims to report the latest research advances in the synthesis and characterization of nanostructured materials and their catalytic applications, in which 10 research articles on the catalytic reaction studies using various nanostructured catalysts were contributed [1,2,3,4,5,6,7,8,9,10].

Novel metal nanoparticles are supported rigidly on the nanoporous metal oxides with high surface areas, which can be used as highly active and selective heterogeneous catalysts. For example, Au nanoparticles were supported on CeO_2_-Mg(OH)_2_, by which strong metal support–interaction (SMSI) could be generated at the interface of Au nanoparticles and metal oxides [1,2]. The SMSI is the special interaction existing at the interface between electron-rich metal nanoparticles and metal oxides, which changes the electronic properties of metal surfaces that can act as the main catalytic sites [11,12,13]. Reaction results such as catalytic activity, reaction rate, and product selectivity can be changed due to SMSI. SMSI can be controlled by the relative composition of CeO_2_ and Mg(OH)_2_, resulting in the catalytic activity during the oxidative esterification of aldehydes with alcohols to produce alkyl esters [2].

Bimetallic Pt-Mo catalysts were synthesized by adding supports to the carbon surface. Compared with the SMSI occurring at the interfaces of metal species, the interaction between the metal and the carbon surface is relatively weak. However, the formation of strong Mo-C bonds enhances the surface dispersion of Pt close to the Mo [3]. Such Pt-Mo/C catalysts engineered in a nanoscale with controlled compositions exhibited remarkable differences in activity and selectivity during the hydrogenation of cinnamaldehyde where the chemoselective hydrogenation is one of the most important issues [3]. As another example of selective hydrogenation, Ag nanoparticles were used by constructing core–shell-structured Ag@SiO_2_ nanospheres for use as the robust heterogeneous catalyst [4]. The Ag@SiO_2_ core–shell catalyst was prepared in one-pot synthesis, which exhibited extremely high catalytic activity in the hydrogenation of nitrobenzene with 99.9% conversion and 100% selectivity to aniline [4]. This core–shell structure has long been attracting a great deal of attention as the stable heterogeneous catalyst that can accommodate metal nanoparticles inside the stable hollow metal oxide sphere with a porous shell for providing facile diffusion pathways to the reactants and products [4,14]. Recently, the solid-state pseudomorphic transformation of colloidal silica nanospheres was also developed to easily and efficiently convert the dense silica wall to the porous silica shell, in which the synthesis can be generalized further to make novel metal-nanoparticle-encapsulated mesoporous silica nanospheres [14].

In addition to the novel metal nanoparticles, transition metal nanoparticles could be supported with various catalytic compositions for hydrogenation of CO_2_ as the CO_2_ utilization technology [5,15]. Ni-based heterogeneous catalysts were synthesized by melting-assisted preparation method, which exhibited a high catalytic activity in the CO_2_ methanation process [5]. Similarly, it was reported that the activity and product selectivity in CO_2_ hydrogenation process could be affected by the design of nanostructured catalytic materials, in which the proximity and surface composition of main catalytic components on the nanostructured CuAl_2_O_4_ played a crucial role in producing liquid-phase C_5+_ hydrocarbons with upscaling productivity [15].

The surface and composition controlled nanostructured materials could be used in photocatalysis [6,7,8]. Tailored synthesis of photoactive TiO_2_ nanostructures were used as the photocatalysts in water splitting for H_2_ production [6,7] and photodegradation of 4-chlorophenol [6]. The morphology-controlled TiO_2_ on which the surface was decorated with Ag nanoparticles exhibited different photocatalytic activities [6]. Nanorod-shaped TiO_2_ supporting Cu possessed a narrower bandgap than commercial TiO_2_, which exhibited better photocatalytic activity during water splitting [7]. Bio-derived synthesis of carbon dots from fish scales opened an interesting approach to synthesizing photocatalytic nanomaterials that could be used for the single-electron photoreduction in methyl viologen [8].

Ordered nanoporous materials were used as the versatile heterogeneous catalysts itself or the support for metal nanoparticles to make multifunctional catalysts [9,10,16,17]. Metal–organic frameworks (MOFs) have remarkably wide structural versatility in controlling the sizes, shapes, and functional groups [9]. The stable UiO-66 MOF was used as a separator to make an interparticle space that promoted mass transport and enhanced cyclability during the charge/discharge process in Li-S batteries [9]. The ordered mesoporous silica SBA-15 was used as a sacrificial hard template to prepare Fe-N-C as the non-precious metal catalysts [10]. The synthesized Fe-N-doped ordered mesoporous carbon activated by phosphoric acid was used in electrochemical oxygen reduction reaction (ORR), where the ORR activity was controlled by pore structure of the catalyst and P doping. Zeolite is one of the representative ordered nanoporous materials, which could be used for supporting various metal nanoparticles inside the micropore cavity or various metal cations bound to the electronegative aluminosilicate framework [16,17]. The Pd metal-nanoparticle-supported mesoporous zeolites exhibited very fast dehydrogenation kinetics during H_2_ evolution from the LOHC compound, which could be attributed to the enhanced diffusivity through its mesoporous structure and the high density of external acid sites on the mesopore wall [16]. The transition metal-cation-exchanged zeolites were used for CH_4_ chlorination with Cl_2_ and exhibited nearly 100% selectivity CH_3_Cl as a desired product, which could be attributed to the controlled electrophilic catalytic sites originating from the transition metal cations bound to the zeolite framework [17].

A large amount of research regarding the synthesis of nanostructured materials, including size/shape-controlled metal nanoparticles, morphology-controlled metal oxides, and structure-controlled nanoporous materials, has been discussed in this Special Issue, communicating with other relevant research advances reported recently. The nanostructured catalytic materials can be used not only for manufacturing value-added products, but also for making human society a more sustainable place.

Finally, we sincerely acknowledge all valuable contributors, as well as the editorial teams of *Nanomaterials*. We hope that this Special Issue inspires many worldwide researchers in the synthesis of nanostructured materials for use as highly active, selective, and robust heterogeneous catalysts.
